# Fitness of Outbreak and Environmental Strains of *Escherichia coli* O157:H7 in Aerosolizable Soil and Association of Clonal Variation in Stress Gene Regulation

**DOI:** 10.3390/pathogens3030528

**Published:** 2014-06-30

**Authors:** Subbarao V. Ravva, Michael B. Cooley, Chester Z. Sarreal, Robert E. Mandrell

**Affiliations:** Produce Safety and Microbiology Research Unit, United States Department of Agriculture, Agriculture Research Service, Western Regional Research Center, Albany, CA 94710, USA; E-Mails: michael.cooley@ars.usda.gov (M.B.C.); chester.sarreal@ars.usda.gov (C.Z.S.); remandrell@gmail.com (R.E.M.)

**Keywords:** *Escherichia coli* O157:H7, curli, stress-response genes, environmental fitness, MLVA, adaptation, *rpoS*, *slp*, *osmY*, *hdeA*, *nemR*, *marR*, mutations

## Abstract

Airborne dust from feedlots is a potential mechanism of contamination of nearby vegetable crops with *Escherichia coli* O157:H7 (EcO157). We compared the fitness of clinical and environmental strains of EcO157 in <45 µm soil from a spinach farm. Differences in survival were observed among the 35 strains with *D*-values (days for 90% decreases) ranging from 1–12 days. Strains that survived longer, generally, were from environmental sources and lacked expression of curli, a protein associated with attachment and virulence. Furthermore, the proportion of curli-positive (C^+^) variants of EcO157 strains decreased with repeated soil exposure and the strains that were curli-negative (C^−^) remained C^−^ post-soil exposure. Soil exposure altered expression of stress-response genes linked to fitness of EcO157, but significant clonal variation in expression was measured. Mutations were detected in the stress-related sigma factor, *rpoS*, with a greater percentage occurring in parental strains of clinical origin prior to soil exposure. We speculate that these mutations in *rpoS* may confer a differential expression of genes, associated with mechanisms of survival and/or virulence, and thus may influence the fitness of EcO157.

## 1. Introduction

Five percent of all produce associated outbreaks during 1998 to 2007 were linked to *Escherichia coli* O157:H7 (EcO157) and 19% of all EcO157 associated outbreaks were due to consumption of contaminated produce [1]. Pathogens attached to contaminated “ready to eat” produce are difficult to remove [2]. Investigations of major outbreaks associated with produce occurring in the US and in other parts of the world indicate that pre-harvest contamination in the field occurs. [3]. Controlling pre-harvest contamination will require an understanding of the biological and environmental factors that regulate the proliferation and survival of pathogens during their transport from animal reservoirs to produce.

In complex and rapidly changing environments, survival is dependent on pleiotropy and plasticity of the genome. Even in simple laboratory environments, thousands of mutations can occur and have been shown to lead collectively to improvements in fitness [4]. Furthermore, as adaptive mutations occur in complicated regulatory networks, the pleiotropy inherent in the genome can result in a wide variety of effects [5,6,7]. There are numerous habitats, in and around growing produce, each of which may affect differentially the survival and virulence of EcO157. EcO157 survives for extended periods in protected environments of soil and manure [8,9] and to lesser extent in water [10,11], but it is very sensitive to decreased moisture [12,13] and oxygen [14], to high temperature [15,16], acidity [17] and UV levels [18]. Therefore, it is probable that most EcO157 cells exposed to stressful environments outside the animal host fail to survive [13]. Nevertheless, survival in water and soil can be sufficient to allow transport of the pathogen by surface water, irrigation and wind, possibly leading to produce contamination.

Survival of EcO157 in sufficient numbers to cause clinical infection involves often the formation of biofilms, facilitated by the production of adhesins and exopolysaccharides [19,20]. One such adhesin, curli, along with the production of cellulose, has been shown to enhance bacterial adherence necessary for the formation of biofilms and favor host colonization [21,22]. Curli are thin aggregative fimbriae and act as a virulence factor by promoting attachment to eukaryotic cells [23,24]. Curli fimbriae, encoded by *csgA*, are expressed in response to low temperature, low oxygen, low osmolarity, and nutrient limitation [25].

Consistent with the role of curli in biofilm formation, curli mutants may confer a selective advantage, possibly allowing rapid adaptation to acidic environments [26]. This appears to occur by maintenance of divergent curli subpopulations even in the absence of selection. However, the acid adaptation in the curli mutants was not due to changes in *CsgA*, indicating that mutations affecting curli are pleiotropic and possibly occur elsewhere in the regulatory network controlled by the alternate sigma factor RpoS, a general regulator for stress factor genes in *E. coli* [27,28,29,30]. This sigma factor controls genes for oxidative stress, osmotic stress, acidification, detoxification, and antimicrobial resistance [31,32]. However, studies have shown that *rpoS* mutants accumulate in prolonged stationary phase cultures [33,34], suggesting that a loss in expression of this important regulator may, under some conditions, confer a selective advantage [35].

In the present study, we selected a diverse collection of EcO157strains to test the hypothesis that adaptive mutations in rpoS may occur at differential rates. The tested EcO157 included strains that were closely-related phylogenetically to the 2006 spinach outbreak as well as another set of strains with diverse phylogeny. The strains were inoculated into fine soil that could be aerosolized by light wind conditions. The differential survival, gene expression and *rpoS* mutations observed were described.

## 2. Results and Discussion

### 2.1. Survival of EcO157 Strains in Moist Soil

**Figure 1:**
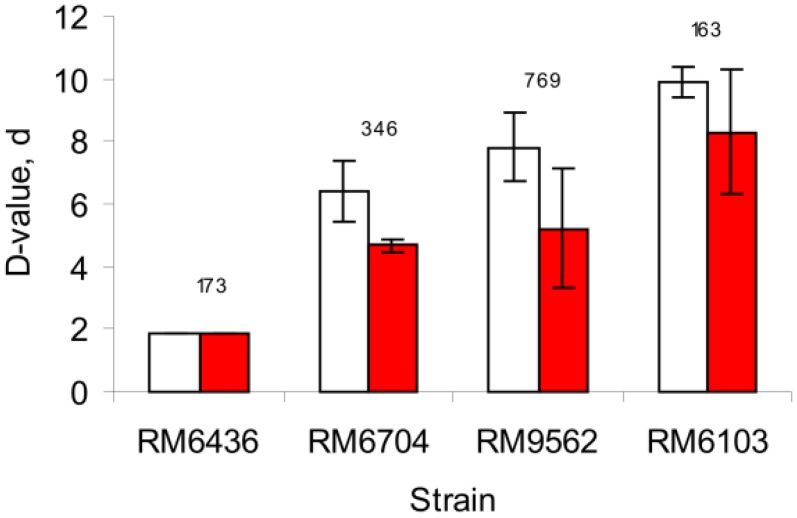
Fitness of EcO157 strains of different Multi-Locus Variable-number tandem-repeat Analysis (MLVA) types isolated from cow feces in soil. Survival of EcO157 was monitored in soil with (white) or without (red) maintaining the moisture at 50% water holding capacity during a 14-day incubation period. The numbers representing the MLVA sequence types are noted above the bars. Standard deviations of *D*-values are shown for triplicate treatments.

To examine the fitness of EcO157 strains with different Multi-Locus Variable-number tandem-repeat Analysis (MLVA) sequence types isolated from cow feces, the survival of EcO157 strains was studied in moist soil. Incubation of PBS-washed cells of RM1484 in soil at 25 °C resulted in a rapid decrease (99.99%) from 10^8^ CFU per gram of dry soil to 10^5^ CFU within 2 h of inoculation. The moisture level in these initial soil treatments from the inoculum was 10% by weight and equated to 19% water holding capacity (WHC). Thus, subsequent comparisons on the survival of EcO157 strains were done in soils adjusted to a moisture level of 50% WHC. Maintaining moisture during incubation increased survival as indicated by higher *D*-values, which represented the number of days for 90% reduction in EcO157 populations. As shown in Figure 1, the survival of all cow fecal strains resulted in increased *D*-values (*p* = 0.005), but the increase was proportional (*r^2^* = 0.803, *p* <0.0001) to the treatments without moisture adjustment during incubation. However, EcO157 strain RM6704 decreased more rapidly with a *D*-value of 4.7 ± 0.2 d (*p* < 0.05) in soil without moisture maintenance compared to 6.4 ± 1.0 d in moisture-maintained soil. Although moisture decreased by 50% in non-maintained soils, the soil still remained moist as evidenced by the ability to shape “balls” by squeezing firmly the soil by hand during the incubations. The source of variation due to strain differences was significant (*p* < 0.0001) whether moisture levels were maintained or not. Therefore, fitness of EcO157 strains in soils was compared at an initial level of 50% WHC, but without adjusting the moisture during incubations.

### 2.2. Fitness of EcO157 Strains in Soil Initially Adjusted to 50% WHC

*D*-values for EcO157 in soils varied for strains isolated from different environmental sources and from different Multi-Locus Variable-number tandem-repeat Analysis (MLVA) types (Table 1). Seventy-five percent of the clinical strains obtained from patients in different states and linked to the 2006 spinach outbreak (MLVA 163) decreased similarly in numbers with a *D*-value of 6.8 ± 0.6 d. In contrast, *D*-values were higher and ranged from 8.3–12.3 d for strains of the same MLVA type isolated from bagged spinach (RM6067) and from wild pig and cow feces (RM6155, RM6103). Similarly, environmental strains originating from samples of pasture soil (RM6149, RM9834, RM9853, RM9854) and river water (RM6102) resulted in survival with *D*-values > 8 d. EcO157 strains with a 100% C^−^ phenotype (Table 1) survived significantly longer than the clinical strains.

**Table 1:** Fitness of clinical and environmental strains of EcO157 in fine soil.

Strains ^a^	Origin	MLVA Type	Curli Proportion, %	*D*-value	Source
RM6331	Human	163	0	7.5	2006, Oregon State Public Health Lab, spinach outbreak, OR
RM6653	Human	163	80	7.1	2006, CDC, spinach outbreak, WI
RM6654	Human	163	50	6.1	2006, CDC, spinach outbreak, NM
RM6657	Human	163	70	6.1	2006, CDC, spinach outbreak, UT
RM6663	Human	163	30	6.4	2006, CDC, spinach outbreak, PA
RM6069	Human	163	100	1.4	2006 spinach outbreak, PA [36]
RM11780	Human	964	0	7.4	2010, Oahu, Korean Barbecue, HI
RM11784	Human	965	0	10.8	2010, Oahu, Korean Barbecue, HI
RM6067	Spinach	163	50	10.3	2006 spinach outbreak, PA [36]
RM6155	Pig feces	163	0	12.3	2006, Ranch A pasture, CA
RM6101	Pig feces	176	0	10	2006 spinach outbreak, CA [36]
RM6102	Water	176	0	8.9	2006 spinach outbreak, CA [36]
RM9854	Soil	792	0	9.5	2009, Ranch J, CSREES ^b^ study, CA
RM9834	Soil	778	0	10.8	2009, Ranch J, CSREES study, CA
RM9853	Soil	778	NT^c^	8.4	2009, Ranch J, CSREES study, CA
RM6149	Soil	176	NT	11.7	2006, Ranch A pasture soil/dust, CA
RM1484	Apple juice	23	100	6	1996, FDA, apple juice outbreak, CA
RM6103	Cow feces	163	0	8.3	2006 spinach outbreak, CA [36]
RM6107	Cow feces	176	0	10.8	2006, Ranch A pasture, CA
RM6436	Cow feces	173	0	1.9	2006 spinach outbreak, CA [36]
RM5038	Cow feces	15	NT	9	2005, Salinas, CA
RM10910	Cow feces	881	NT	8.5	2009, CSREES study, CA
RM6704	Cow feces	346	NT	4.7	2007, Sierra Foothills, Browns valley, CA
RM7024	Cow feces	416	NT	6.8	2007, Sierra Foothills, Browns valley, CA
RM6121	Cow feces	187	NT	11.5	2006, Ranch J, replacement heifers, CA
RM7354	Cow feces	186	NT	9.5	2007 Leafy green outbreak (suspected), HI
RM7469	Cow feces	534	NT	6.1	2008, CSREES study, CA
RM9562	Cow feces	769	NT	5.2	2009, CSREES study, CA
RM6666	Cow feces	352	NT	6.2	2007, Sonoma dairy, CA
RM7437	Cow feces	489	NT	7.5	2008, CSREES study, CA
RM8436	Cow feces	490	NT	6.2	2008, CSREES study, CA
RM7438	Cow feces	486	NT	5.1	2008, CSREES study, CA
RM6009	Moore swab	158	NT	5.6	2006 spinach outbreak, CA [36]
RM5686	Moore swab	89	NT	5.7	2006 spinach outbreak, CA [36]
RM5724	Moore swab	143	NT	4.3	2006 spinach outbreak, CA [36]

^a^ Produce Safety and Microbiology Research Unit, culture collection maintained by Anne Bates. ^b^ 2007 *Cooperative State Research, Education, and Extension Service (CSREES) research grant* “Ecology and epidemiology of *Escherichia coli* O157:H7 in fresh produce production regions on the Central California Coast”, *U. S. Department of Agriculture*. ^c^ NT; Not tested for proportion of curli variants.

### 2.3. Survival of Curli Variants in Soil

**Figure S1:**
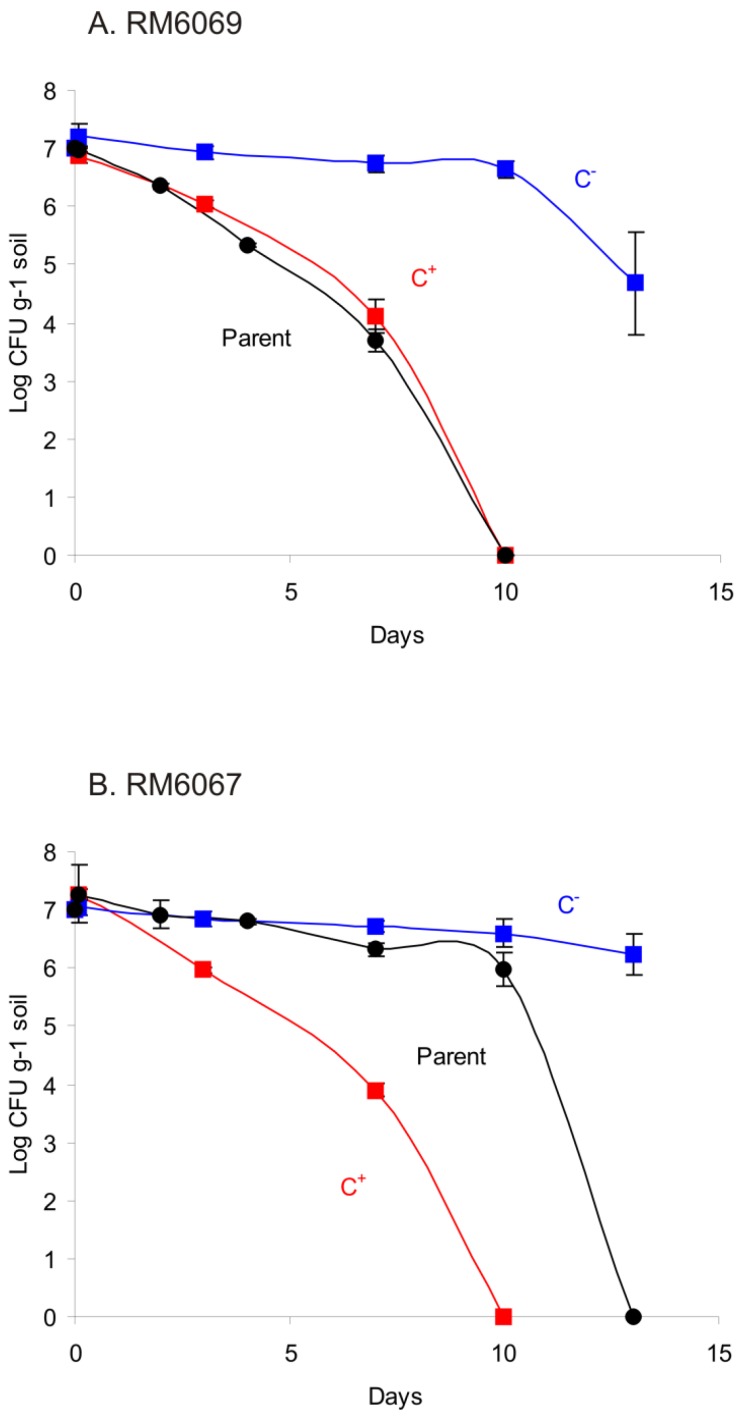
Fitness of curli variant subpopulations of EcO157 strains in soil. Survival of C^−^ (blue ■) and C^+^ (red ■) variant subpopulations of a clinical strain (RM6069) and a spinach strain (RM6067) linked to 2006 spinach outbreak. The decreases in curli variant subpopulations were compared with that of the parent (●) EcO157 strains. Both parental strains are of the same MLVA type 163, but the curli variants typed as different, but highly related, MLVA types (see Table 2).

Since C^−^ strains gave higher *D*-values in the previous test, C^−^ and C^+^ variant clones of four EcO157 strains showing the mixed curli phenotype were tested. C^−^ variants of a strain linked with the apple juice outbreak and three with the 2006 spinach outbreak survived longer compared to parental strains or the C^+^ variants in soil, regardless of MLVA type (Table 2). The kinetics of the decreases of curli variants in soil as compared to parental strains RM6069 and RM6067 are shown in Figure S1. Consistent with the results noted above, the parental strain RM6067 with 50% “C^−^ phenotypes” survived longer than parental strain RM6069 with a “C^+^ phenotype” (nearly 100% C^+^; Figure S1). Further analysis of curli variant strains RM6067 and RM6069 revealed minor tandem repeat (TR) differences. For the curli variant strain RM6069 there was one TR difference at Vhec4 locus for C^−^ variants and for strain RM6067, there were two TR differences at Vhec1 locus for C^+^ variants and one TR difference at Vhec1 for C^−^ variants. These TR differences resulted in a change in MLVA sequence type assignment (Table 2).

**Table 2:** Fitness of curli variant clones as compared to the parental strains of EcO157 linked to apple juice and spinach outbreaks.

Strain	Origin	MLVA Type ^a^			*D*-value, d ^b^		
		Parent	C^+^	C^−^	Parent ^c^	C^+^	C^−^
RM1484	Apple juice	23	23	23	6.0 ± 0.3	5.4 ± 1.4	10.1 ± 2.9
RM6069	Human	163	163	361	1.4 ± 0.0	1.4 ± 0.0	6.1 ± 2.0
RM6067	Spinach	163	975	164	10.3 ± 2.5	1.4 ± 0.0	20.2 ± 11.0
RM6103	Cow feces	163	163	163	12.5 ± 1.3	4.8 ± 1.2	19.9 ± 10.8

^a^ MLVA differences between C^+^ and C^−^ variants represent one or two tandem repeat differences at a single locus. ^b^ Values are averages (± standard deviations) of triplicate treatments. ^c^ See Table 3 for proportion of C^+^ variants in parental strains associated with 2006 spinach outbreak.

### 2.4. Survival of EcO157 in Soil after Three Successive Transfers in Soil

Based on the observation that curli variants differed in their ability to survive in soil compared to the parental strains (Table 2), we tested whether increased exposure to soil altered the curli phenotype distribution in the survivors. The results indicated that prolonged survival in soil was associated with a shift towards lack of curli expression (Table 3). C^+^ variants surviving during the three repeat passages resulted in rapid decreases compared to C^−^ variants after the third pass. Continued exposure to the soil resulted in a decreased ratio of the C^+^ to C^−^ variant population. Furthermore, in those strains initially lacking C^+^ variants, no C^+^ variants were found after three successive passages in soil.

**Table 3:** Fitness of environmental and clinical EcO157 strains linked to 2006 spinach outbreak after three successive transfers to fresh soil from outbreak leafy green field.

Strain ^a^	Origin	State	*D*-values ^b^		C^+^ variants, %	
			5 d	18 d	Parent ^c^	Post Exposure
RM6069	Human	PA	7.0	2.6	100	90
RM6653	Human	WI	7.1	4.5	80	50
RM6654	Human	NM	6.1	7.6	50	10
RM6663	Human	PA	6.4	8.2	30	0
RM6331	Human	OR	7.5	7.6	0	0
RM6155	Pig feces	CA	7.9	8.4	0	0
RM6103	Cow feces	CA	9.8	7.1	0	0
RM6067	Spinach	CA	6.0	8.2	50	0
RM9834	Soil	CA	8.9	7.3	0	0

^a^ All strains are of MLVA type 163 except for RM9834 (type 778). ^b^ Survival of EcO157 in soils inoculated with pooled organisms surviving from the previous soil incubations. Five-day and 18-day *D*-values are those after the first and third incubation in soil, respectively. After each passage through soil, all presumptive EcO157 colonies were pooled and transferred to a fresh batch of soil. ^c^ We were able to isolate a few curli variants from parental strains designated as 100% or 0% curli.

### 2.5. Expression of Selective Stress Genes of Curli Variants that Survived Three Passes in Soil

**Figure 2:**
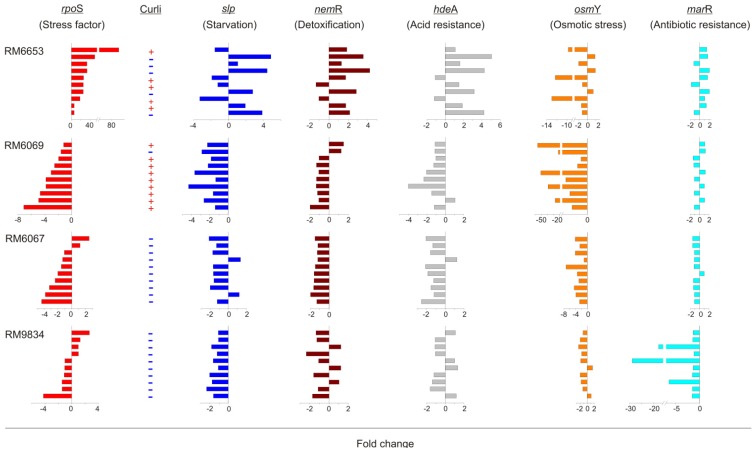
Clonal variation in expression of selective stress genes in EcO157 strains. Fold change in the expression of stress factors for each of 10 subcloned curli variants that survived three successive exposures to soil were plotted relative to expression in the parental strains RM6653, RM6069, RM6067 and RM9834. Clonal variation in expression of *rpoS* and the corresponding curli type of the subclones are shown for comparison.

Gene expression of stress-related genes and curli phenotype was evaluated with four EcO157 strains exposed to three passes through soil (Figure 2 and Table S1). For clinical strains RM6653 and RM6069 linked to the 2006 spinach outbreak, several of the surviving clones retained the C^+^ phenotype after soil exposure. In contrast, clones of RM6067, environmental isolates from spinach, all lost C^+^ phenotype. The phenotype of strain RM9834 was C^−^ and remained C^−^ in the surviving clones. Considerable clonal variation was seen with each of the six stress-related genes. The greatest curli variation was seen from the clones of strain RM6653, which also showed the largest up-regulation of the sigma factor *rpoS.* In strain RM6653, there was a negative relationship between curli expression and expression in *slp*, *nem*R, *hde*A and *osm*Y, with strong up or down regulation in C^−^ or C^+^ variants, respectively. This relationship is also seen in strain RM6069, where only one clone was C^−^. However, strains RM6067 and RM9834, which are all C^−^ variants show a predominantly reduced down-regulation in the same genes. Expression in *marR* showed a very modest positive relationship with the curli phenotype, in that expression increases or decreases only approximately two-fold in the C^+^ or C^−^ clones, respectively, in RM6653, RM6069 and RM6067. In the clones of RM9834, expression of *mar*A was still positively related with curli, but two of the clones (3, 5) show substantially larger down regulation.

### 2.6. Clonal Variation in rpoS Gene Sequence of EcO157 Strains

**Figure 3:**
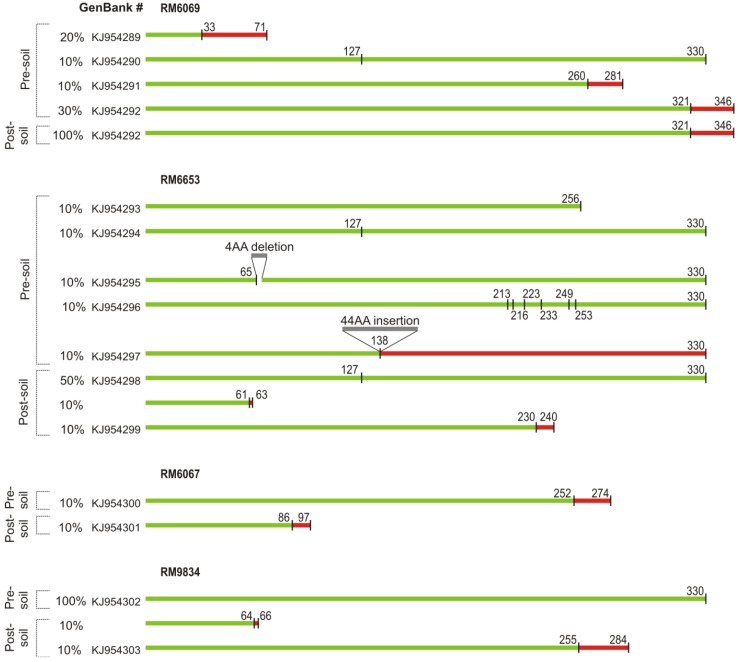
Predicted protein structure from *rpo*S sequence from selected parental or post-soil exposure strains. *rpo*S coding regions from 10 colonies each from both parental and post-soil exposed strains were sequenced. Predicted amino acid (AA) sequence corresponding to the rpoS protein of EDL933 (330 AAs) are indicated in green. Frameshift mutations causing non-synonymous AA substitutions are indicated in red. Position of mutations (insertions, deletions or point mutations) and stop codons are indicated above the lines, relative to the EDL933 RpoS sequence. Percent clones showing mutations and GenBank accession numbers for sequences are also shown.

The clonal variation in the expression of *rpoS* gene, shown in Figure 2, stimulated us to determine whether there were mutations in the *rpoS* gene from these clones. The sequence of *rpoS* gene in 10 clones pre- and post-soil exposure for each of four O157 strains was compared with the *rpoS* sequence of genome strain EDL933 [37]. No *rpoS* mutations were found in pre-soil clones from the soil strain RM9834, but two mutants were found in the 10 post-soil clones from RM9834 and both mutations will produce frameshifts giving pre-mature stop codons as indicated in Figure 3. Similar truncated proteins were predicted from sequence from clones of the other three strains. In fact, at least one of the ten clones (both pre- and post-soil) sequenced from the other three strains indicated a significant truncation. Point mutations leading to transversions and in-frame deletions and insertions were also found in RM6069 (pre-soil) and RM6653 (pre- and post-soil) clones. Analysis of strain RM6653 resulted in one pre-soil clone with six point mutations (both transitions and transversions). For strain RM6069, three pre-soil clones and 10 post-soil clones with an 11 bp deletion 27 bp from the 3' end which created a frameshift with a stop codon 75 bp downstream, resulting in full-length protein with an additional 25 amino acids at the carboxyl-terminus. The number of pre- or post-soil clones that would be expected to produce a non-functional RpoS protein was much higher in the pre-soil clinical clones from strains RM6069 and RM6653. Furthermore, all post-soil clones from the clinical strains that retained the C^+^ phenotype were expected to be RpoS^+^.

### 2.7. Discussion

Numerous studies have reported that enteric human pathogens, including EcO157, shed in the feces of animals can survive for extended periods of time [3]. EcO157 strains that can adapt to the stressful conditions in pasture and/or feedlot soil (e.g. low moisture and nutrients, UV) and may survive in EcO157-laden soil particles that can become airborne and transported from point sources (e.g. feedlots) to produce fields [38]. We analyzed a collection of different EcO157 strains isolated from human and environmental samples to determine their fitness in conditions conducive to aerosolization and characterized selected stress-related genes in population variants that survived in soil fraction <45 μm. 

Strain variation of fitness of EcO157 in soil was observed as indicated by *D*-values ranging from 1.4–12.3 d for 35 strains. EcO157 strains isolated originally from cow feces and of different MLVA genotypes differed in survival *(D*-value of 2–12 d). This suggests that any specific fitness characteristic of EcO157 varies over a wide range depending upon genetic differences. It should be noted, however, that the influence of soil physical, chemical and biological factors [14,39,40,41] could complicate asserting genotype differences as primary factor in soil fitness. Nevertheless, due to the extensive processing of the soil used in this study, all strains were subjected to the same abiotic factors. Therefore, our study indicated that various EcO157 strains have different fitness capabilities related to the fine soil conditions conducive to dispersal in aerosols.

Some of strains we analyzed for fitness in soil were initially isolated during the 2006 spinach outbreak investigation from wild pig feces, cow feces, soil and water and were indistinguishable and/or highly related to human outbreak strains by PFGE and MLVA [36,42]. This set of highly related strains from diverse sources provided an opportunity to compare the fitness capabilities of strains under relevant environmental conditions. It is noteworthy and relevant to these soil experiments that we had isolated the EcO157 strain (RM9834; Table 1) from the <45 µm portion of a naturally contaminated dry pasture soil repeatedly during a 45-day period. Hence RM9834 had exhibited significant fitness in soil prior to these experiments. In contrast, apple juice outbreak strain RM1484 resulted in a 99.9% loss of culturability within 2 h after inoculation to relatively dry soil and no cells could be cultured even by enrichment methods after 2 days. These results indicate that EcO157 strains possess highly variable fitness capabilities depending probably on the variable genotypes and resulting phenotypes in the population of cells selected under stressful conditions.

It is logical to assume that the decrease in available water [12] and dehydration [41] are linked to the rapid decreases of EcO157 cells in soil in this study, however, the isolation of EcO157 strains from nearly-dry field soil indicate the presence of at least some cells [43] with superior fitness traits. We expect that these EcO157 cells will survive under very stressful conditions in agricultural environments. Indeed, EcO157 could be isolated even after 86 days from soil microcosms [43]. 

Increased survival of naturally occurring C^−^ variants, originally from environmental sources (Table 1) suggests that prior survival under austere conditions has evolved the ability to survive similar environments in the future [44] and selection pressures imposed by soil environment has a significant impact on population structure [45]. This prediction is consistent with our results showing that C^−^ variants survived significantly longer than the C^+^ variants (Table 2) and repeated passage through soil increased the frequency of C^−^ variants in the survivors, presumably, as a result of rapid die-off of C^+^ subpopulations that are less adapted to stressful conditions in soil. These results indicate that the survival of select phenotypes provides a fitness for some cells to perpetuate under stressful environmental conditions similar to dry feedlots nearby produce fields and/or in non-irrigated dry soils between cropping seasons, or until a suitable niche for re-growth occurs.

Some EcO157 strains exhibited low or no capability of producing C^−^ variants. Clinical strains RM6069 and RM6653 gave a high percentage of C^+^ variants, resulting in low survival, even after repeated exposure of surviving populations to fresh soil (Table 3). In contrast, both environmental and clinical strains, having a high ratio of C^−^ to C^+^ variants, survived better on three exposures in soil and remained C^−^ at the end of the experiments. These results indicate that the fitness of EcO157 strains in soil is reflective of the curli phenotype. Enrichment of C^−^ variants may result from the acidic nature of soil (pH 5.4) used in these fitness studies, consistent with the significant acid stress-resistance reported earlier [26] for C^−^ variants of one of the same strains used in this study. Acid stress resistance is critical for EcO157 survival in the stomach of ruminants and humans, and perhaps other environments, and it is intriguing that, generally, environmental strains survive acid stress much better than the isolated clinical or food strains associated with outbreaks [17]. However as mentioned, acid resistance is not due to expression of *csgA* and is likely due to pleiotropic effects within the general stress response controlled by RpoS. Thus, the enrichment of C^−^ variants in soil may be the result of a combination of stress factors. The environmental strains analyzed in this study have the capability to survive acid and other stress conditions in a soil environment, thus prolonging the stationary phase until favorable environmental conditions appropriate for regrowth induce a break in dormancy.

It was reasonable to presume that altered fitness of individual clones after prolonged soil exposure was also supported by differential gene expression of stress related genes. For example, genetically indistinguishable clinical strains (RM6653 and RM6069) post-soil exposure resulted in 50% and 10% C^−^ variants for RM6653 and RM6069, respectively, correlating with improved fitness of RM6653 compared to RM6069. These same variants of RM6653 exhibited significant upregulation of *rpoS* along with other stress-related genes, *osmY* not included (Figure 2). In contrast, a food strain (RM6067, spinach) and an environmental strain RM9834 (soil), both of similarly high fitness prior to, or after, soil exposure, correlated with a predominance of C^−^ variants, and downregulation of most stress responsive genes in a majority of clones (>80%). These results reflect an inverse relationship between the expression of curli and the stress–related genes we analyzed in this study.

Osmotic stress gene *osmY* was down-regulated in majority of the clones (88%) for all 4 strains exposed to soil, but significantly down-regulated in C^+^ subclones from both clinical isolates. These results are similar to published results using two of the same strains in the current study, RM6069 and RM6067, where the clinical strain showed much stronger down regulation of *osmY* compared to the environmental strain. These types of genetic polymorphisms with alterations of expression of *rpoS* and *osmY* are consistent with those measured for EcO157 strain EDL933 (1982 hamburger outbreak) [35].

We determined that *rpoS* mutations occurred predominantly in the clinical strains (Figure 3) with 30%–50% of the pre-soil clinical strains with mutations that either alter or eliminate highly conserved regions of the protein necessary for binding to the core holoenzyme or recognition of the −10 and −35 promoter sequences. Mutational changes in *rpoS* most often happen in environments where fitness does not depend on stress resistance, but requires transcription of vegetative genes for maximal growth, such as the high nutrient conditions within the gut [46]. These phenotypes typically fail to survive (Table 2) under stressful environmental conditions [47]. Consistent with this, mutations in *rpoS* occurred, though less frequently, in the pre-soil spinach strain RM6067 and not at all in the soil strain RM9834.

Mutations found in rpoS in subclones after three passages through soil were more uniformly distributed between clinical and environmental strains. Twenty percent of the clones from the soil strain RM9834 were mutants which truncated the RpoS. One hundred percent of the post-soil clones of the clinical strain RM6069 showed an 11 bp deletion, but is carboxyl-terminal to the −35 binding region and would be expected to be functional. Likewise, 50% of the post-soil clones of the clinical strain RM6653 showed a transversion at position 127 of RpoS, causing substitution of asparagine for isoleucine, but is in a poorly conserved region NH_3_-terminal to the rpoD box is not likely to produce a non-functional protein [48]. Nevertheless, 10%–20% of post-soil clones of RM6653, RM6067 and RM9834 contained mutations in *rpoS* that will produce significantly truncated proteins (Figure 3). Thus, 87% of the post-soil clones are expected to carry a functional *rpoS* gene. A shift towards higher percentage of clones with functional RpoS was expected, since all clones had passed 3 times through the same stressful soil environment. Even greater percentages of functional RpoS would be expected with subsequent passages, along with greater numbers of C^−^ variants.

The results we have presented indicate that stress- and virulence-related gene expression corresponding to the C^−^ phenotype was well suited to fitness in the soil environment and particularly particles <45 µm. As such, it is fit for survival in airborne dust and transport to nearby vegetable crops. It will be informative to evaluate prevalence of the curli phenotype with repeated passage on produce. Strains isolated from bagged-spinach linked to the 2006 outbreak had similar gene expression profiles as the clinical strains and also contained a high proportion of C^+^ subclones [49]. However, exposure of EcO157 strains to romaine lettuce or lettuce extracts, were reportedly more virulent, based on increased stress and virulence gene expression [31,50]. Understanding the factors related to EcO157 survival in the leafy vegetable production environments is critical to developing intervention strategies that can minimize the probability of an outbreak associated with leafy vegetables.

## 3. Materials and Methods

### 3.1. Soil used in EcO157 Fitness Studies

Soil collected from a produce field from lower Salinas Valley in Monterey County, CA, USA (Farm R) [36] was used for EcO157 strain fitness studies. Soil particles <45 µm (US standard sieve, 325 mesh, Hogentogler, Columbia, MD, USA) were selected from dry soil by sieving through sequentially smaller mesh screens. This soil fraction contained fine particles that would aerosolize most readily. The final soil sample represented approximately 1.5% of the field soil and was used in all the fitness studies.

### 3.2. Strains of EcO157

EcO157 strains for evaluation of fitness in soil (Table 1) were selected based on sample source and genetic similarities determined by MLVA [36]. The purity of cultures was confirmed by plating on Rainbow agar (Biolog, Hayward, CA, USA) containing novobiocin (20 µg/mL, Sigma-Aldrich, St. Louis, MO, USA) and tellurite (0.8 µg/mL, Dynal, Life Technologies, Grand Island, NY, USA) (Rainbow-NT) [36].

### 3.3. Preparation and Enumeration of Strains for Inoculation of Soil

Isolated colonies from Rainbow-NT agar were grown overnight in 10% Luria broth (LB, Fisher Scientific, PA, USA) at 25°C, cells were separated by centrifuging at 10,000 x g for 5 min and washed twice in phosphate-buffered saline (PBS, pH 7.0). The cells resuspended in PBS were adjusted to OD_600_ of 0.3 prior to inoculation to the soils. Enumeration of EcO157 from inoculated soils was carried out by plating 100 µL volumes of 10-fold serial dilutions of soil in PBS onto Rainbow-NT agar and bluish-grey colonies were counted after over-night incubation at 37°C. One-hundred mg soil samples collected at various intervals during the incubations were used to make serial dilutions. Isolated colonies of EcO157 strains prior to or post-soil exposure and curli-variant subpopulations used in gene expression studies were confirmed for the presence of EcO157-specific O-antigen transporter gene rfbE [36].

### 3.4. Fitness of EcO157 Strains in Soil from a Produce Field

One gram samples of <45 µm-soil in 4 mL screw capped glass vials (Wheaton Science Products, Millville, NJ, USA) were inoculated with ~1 × 10^7^ CFU of EcO157 cells and adjusted to moisture at 50% WHC. WHC is 26.1% moisture on dry weight basis. EcO157 cells in 261 µL PBS was added to the soil and mixed thoroughly with a sterile spatula prior to incubations. Vials containing the inoculated soils were capped loosely to allow aeration, incubated at 25 °C and sampled at regular intervals for the enumeration of surviving EcO157 cells as described above. *D*-values were calculated based on the decreases in EcO157 populations during 10–14 days incubation periods.

To delineate the influence of moisture in overshadowing other soil factors, cow feces strains of four different MLVA types were compared to check if strains different phylogenetically also differ in fitness to moisture effects. Two strains linked to the 2006 spinach outbreak (RM6103, RM6436) and two other strains isolated from different feedlots (RM6704 from Browns Valley and RM9562 from Salinas Valley of California) were compared. The comparisons were in triplicate and all treatments were adjusted initially to moisture of 50% WHC. The moisture in one set of treatments was not adjusted during the incubations and was maintained at 50% WHC in a parallel set of treatments by supplementing sterile distilled water (on a weight basis) just before sampling. The moisture loss at each sampling interval during the 14-day incubation was monitored by weighing another set of vials containing un-inoculated soil. Soils were mixed thoroughly with sterile spatulas before sampling for enumeration of EcO157.

Survival of 35 EcO157 strains (Table 1) was monitored in soil initially adjusted to 50% WHC. Clinical and environmental strains linked to 2006 spinach outbreak and used for analysis in this study were selected based on differences in their sources (e.g. state public health labs). These comparisons were not replicated.

### 3.5. Fitness of Phenotypically Variant Subpopulations

C^+^ and C^−^ variants of EcO157 strains linked to the 2006 spinach outbreak and a strain linked to a 1996 apple juice outbreak (RM1484) were isolated as described previously [26], with some modifications. Briefly, dilutions of cells from frozen stocks were checked for purity on Rainbow-NT agar plates initially and the dilutions of growth from Rainbow-NT in PBS were plated on LB agar without NaCl, but supplemented with 40 µg/mL of Congo red dye (Sigma-Aldrich) and 10 µg/mL of Coomassie brilliant blue G (Congo red agar, Sigma-Aldrich). Red (C^+^) and white (C^−^) colonies were isolated and patched on to Congo red agar plates for further characterization. C^+^ and C^−^ variants and the parental strains were characterized by MLVA typing (see below). The variants were grown in 10% Luria broth and the cells were washed in PBS prior to soil inoculations. The parental strain and the curli variants of each strain in triplicates were compared for fitness in soil as described above. Moisture levels were monitored throughout and the surviving organisms were monitored as described above during the 14-day incubation.

### 3.6. MLVA Genotyping of C+ and C− Variant Clones of EcO157 Strains

MLVA of parental strains and curli variant sub-clones was performed by capillary electrophoresis of amplified products of 11 loci (Vhec1-7, O157-17, O157-19, O157-25 and O157-37) in 3 multiplex reactions using fluorescent primers as described by Cooley *et al.* [36,51]. The number of tandem repeats at each locus was calculated and compared in a BioNumerics (v 6.5, Applied Maths, Inc., Austin, TX, USA) database to determine the MLVA type. Allele numbers were assigned according to published methods and with MLVA algorithms in Bionumerics software.

### 3.7. Measurement of Surviving EcO157 Phenotypes during and after Successive Transfers in Soil

Five clinical (RM6069, RM6653, RM6654, RM6663, RM6331) and three environmental (RM6155, RM6103, RM6067) strains of the same MLVA type of EcO157 linked to the 2006 spinach outbreak were compared. The study also included a strain (RM9834) obtained from dry cow pasture soil. Enumeration methods were the same as described above, except that each cycle of incubation lasted for 5–6 days. Colonies of the typical EcO157 morphology were enumerated on Rainbow-NT agar, pooled and re-grown overnight in 10% LB broth. This additional step of selecting colonies was necessary to differentiate the non-specific growth of native soil bacteria from EcO157 cells. The proportion of curli variants was determined by patching 10 isolated colonies from Rainbow-NT agar on Congo red agar. The cells were pelleted, washed and resuspended in 0.01M PBS to an OD_600_ 0.3 and inoculated into a fresh batch of soil. This process was repeated twice and the proportions of curli variants were determined prior to soil inoculation (6th and 13th day) and at the end of the 3rd and final inoculation (18 d total incubation).

### 3.8. Gene Expression by Clones Surviving Soil Exposure

Ten random clones selected after repeated soil exposure of three EcO157 strains (RM6069, RM6653, RM6067) linked to the 2006 spinach outbreak, one soil strain from a cow pasture (RM9834) highly related by MLVA, and triplicate clones from the parental strains, were selected for analysis of expression of selected stress genes after repeated soil exposure. Cultures were grown overnight in 10% LB broth at 25 °C, followed by a 50% dilution in 10% LB, grown for 4 h (OD_600_ ~ 0.6), and pelleted for isolation of total RNA according to the manufacturer’s instructions for Promega SV Total RNA Isolation System (Promega, Madison, WI, USA). Bacterial RNA was then treated with Turbo DNase I (Applied Biosystems/Ambion, Austin, TX, USA) and the absence of DNA was verified by PCR in the absence of reverse transcriptase using primers for *gyrB* (Table S2). Total RNA concentration was determined using a Nanodrop ND 1000 spectrophotometer (Thermo Scientific). Quantitative Reverse Transcription-PCR assays were performed using Brilliant II SYBR green QRT-PCR 1-Step kit (Stratagene, La Jolla, CA, USA) on MxPro 3000P Cycler (Stratagene) using the 2-step cycling program as per the manufacturer’s instructions. Genes involved in osmotic stress (*osmY*), acid resistance (*hdeA*), antibiotic resistance (*marR*), detoxification (*nemR*) and starvation (*slp, rpoS*) were evaluated using primers described previously (Table S2). Each gene expression reaction was performed in triplicates and all reactions were normalized to the expression of the housekeeping gene, *gyrB*. The ratios of gene expression for each of the 10 clones isolated after soil exposure for each strain and the non-soil exposed parent (average of three replicates) were calculated using the Comparative Quantitation Analysis module of MxPro.

### 3.9. Sequencing of RpoS from Clones of EcO157 Surviving Soil Exposure

The same 10 clones of each EcO157 strain isolated after soil exposure and selected for gene expression analysis were used for sequencing the *rpoS* gene (Method S1). Bionumerics software (Applied Maths) was used to detect mutations in the full length sequence of *rpoS* gene from 10 clones of each of four EcO157 strains before and after soil exposure compared to the characterized sequence of EcO157 strain EDL933 (GenBank accession no. AE00517H) [37]. The gene sequences of rpoS proteins from pre- and post-soil exposed clones of EcO157 strains submitted to GenBank under accession numbers KJ954289 to KJ954303.

### 3.10. Statistical Methods

Two-way ANOVA and linear regression analyses (Prism 4.0, GraphPad Software, Inc., San Diego, CA, USA) were performed on *D*-values of EcO157 strains incubated in soil with or with-out maintaining moisture at 50% WHC to determine if fitness of EcO157 strains in soil was masked by moisture effects. Spearman correlation coefficients (*r_s_*) were calculated to determine if the expression of various stress factors correlate significantly with the expression of *rpoS* gene in clones surviving soil exposure.

## 4. Conclusions

We described that the fitness of EcO157 in potentially aerosolizable soil is associated with the fitness characteristics of surviving subpopulations. We focused our study on differences in the curli phenotypes reported previously as critical in biofilm formation, attachment and virulence [25]. Exposure of EcO157 strains to soil results in: (1) selection/enrichment of the C^−^ variants corresponding to a significant decrease or elimination of C^+^ variants in the population; (2) clonal variation in expression of selected stress factors; (3) mutations in *rpoS* gene in the initial parental strains and surviving C^+^ subpopulations. Our results suggest that exposure of different EcO157 produce-associated outbreak strains to the relatively austere environment represented by relatively dry soil will result in die-off at different rates depending upon the population variation reflected by curli, as one example, and perhaps by additional variants not identified in this study. However, the survival of a small subpopulation of highly fit EcO157 cells increases the probability of these variants to become predominant in a stressful environment. We speculate that soil/stress-related fitness is associated with mechanisms of survival on or in leafy greens and/or virulence determinants and that, if so, these strains are the most likely to be associated with outbreaks. Further studies of the mechanisms of mutations in global stress-response regulators and phenotypic variation relevant to the pre- and post-harvest produce production environment are warranted for identifying potentially hypervirulent EcO157 strains.

## Supplementary Information

**Table S1:** Clonal variation in expression of stress genes in EcO157 strains after three successive transfers in fresh soil.

Stress Gene	Percent Clones Downregulated (Range of Fold Change ^a^)			
	RM6653	RM6069	RM6067	RM9834
*rpoS*	0 (6–83)	100 (−7–−1)	80 (−4–3)	60 (−4–3)
*slp*	40 (−3–5)	100 (−4–−1)	80 (−2–1)	100 (−2–−1)
*osmY*	60 (−13–2)	100 (−56–−2)	100 (−7–−1)	70 (−2–2)
*katP*	90 (−5–1)	100 (−2–1)	100 (−3–−2)	80 (−2–1)
*hdeA*	20 (−1–5)	90 (−4–1)	90 (−3–1)	60 (−2–1)
*oxyS*	30 (−2–2)	30 (−2–27)	70 (−2–1)	100 (−3–−1)
*marA*	30 (−1–2)	60 (−2–1)	70 (−2–2)	80 (−2–1)
*marR*	30 (−2–2)	50 (−1–1)	90 (−2–1)	100 (−29–−1)
*nemR*	20 (−1–4)	80 (−2–2)	100 (−2–−1)	70 (−2–1)
*soxS*	20 (−1–3)	40 (−2–2)	60 (−2–2)	100 (−2–−1)
*ahpF*	70 (−3–2)	60 (−2–1)	90 (−2–1)	30 (−1–2)
*gloA*	20 (−2–2)	60 (−3–1)	90 (−3–−1)	80 (−2–1)

^a^ Gene expression results are averages of triplicates for each of 10 clones. Irrespective of strains or stress factors assayed, downregulation was observed with 69% of all clones.

**Table S2:** Primers for gene expression studies.

Gene	Gene Product/Function	Forward/Reverse Primers, 5'→3' (reference)
*ahpF*	Alkyl hydroperoxide reductase, subunit F	GCAGATTCGCCATATTGACG GCCCTGACCAAACTCTTTCC [31]
*gloA*	Glyoxalase I	ACTCACTGGCGTTTGTTGG GACCGGCGTCTTTCTCTTC [31]
*gyrB*	DNA gyrase, subunit B	GCAAGCCACGCAGTTTCTC GGAAGCCGACCTCTCTGATG [31]
*hdeA*	Acid resistance protein	GCTTCTTCTGCCAGTTGTGAGCAA AGCCAGGAAATCTTCACAGGTCCA [31]
*katP*	EHEC catalase/peroxidase	CGGGAAACTTCAGAAACCTC GCCACAGTCTCCTCATCATC [31]
*marA*	Multiple antibiotic resistance	CGAGGACAACCTGGAATCAC TGCGGCGGAACATCAAAG [31]
*marR*	Multiple antibiotic resistance	CGCGGCGTGTATTACTCC GGTTCGGCAACCTTTCTACC [31]
*nemR*	Predicted DNA-binding transcriptional regulator	CCATTACGCCACATATCACC TATCACGGCCATTTTCCAG [31]
*osmY*	Hyperosmotically inducible periplasmic protein	ACGTTGCGACGCTAAAGAA CATGACGGGAAGGGACGT [32]
*oxyS*	Oxidative stress regulator	GAGCGGCACCTCTTTTAACCCTTG CCTGGAGATCCGCAAAAGTTCACG [31]
*rpoS*	RNA polymerase, sigma S (sigma 38) factor	CGCCGGATGATCGAGAGTAA GAGGCCAATTTCACGACCTA [50]
*slp*	Outer membrane lipoprotein, starvation lipoprotein	AACCTGTGGGATTACGGCTATGGT AGGTGTTACCTGACTCACCGCATT [32]
*soxS*	Global transcription regulator for superoxide response	GTCGTCGCAAAAAAATCAGG TGGGAGTGCGATCAAACTG [31]

## Method S1. Sequencing of *rpoS* gene from clones of EcO157 strains surviving soil exposure

EcO157 strains (10 clones of each) after exposure to soil were grown overnight in 10% LB at 25 °C. Cells were centrifuged, washed in PBS and DNA was extracted using Wizard Genomic DNA purification kit (Promega, Madison, WI, USA). DNA concentrations were determined using Nanodrop ND 1000. The *rpoS* gene-specific forward and reverse primers (Forward: 5' CCCATAACGACACAATGCTG and Reverse: 5' GGTAGGACGCTGACGTGTCT) encoding for a 1321 bp product that encompasses the full length sequence (993 bp) of *rpoS* gene were designed by C. Parker (Produce Safety and Microbiology, USDA, Albany, CA, USA) using Primer Premier 5.0 (Premier Biosoft International, Palo Alto, CA, USA) and obtained from Eurofins MWG Operon (Huntsville, AL, USA). Each PCR reaction of 25 µL contained the following: 1X MasterAmp *Taq* PCR buffer, 1X MasterAmp *Taq* Enhancer, 1.5 mM MgCl_2_, 200 µM each DNTP, forward and reverse primers 0.2 µM each, 0.2 U of MasterAmp *Taq* DNA polymerase (Epicentre, Madison, WI, USA), and 50 ng of genomic DNA. Amplifications were performed using a Dyad thermocycler (BioRad, Hercules, CA, USA) using the following parameters: 1 cycle of 3 min at 95 °C, 35 cycles of 30 s at 95 °C, 30 s at 58 °C, and 2.5 min at 74 °C with a final extension at 74 °C for 7 min. PCR products were checked for the presence of 993 bp fragment on 1% agarose gel and the products were purified using DNA Clean and Concentrator-5 kit (Zymo Research, Orange, CA, USA) by using 5 volumes of DNA binding buffer to each volume of PCR product mixture as per manufacturer’s instructions.

Big Dye terminator cycle sequencing kit (v. 3.1, Applied Biosystems, Foster City, CA, USA) was used to perform sequencing reactions using both forward and reverse primers, and Big Dye XTerminator purification kit was used to immobilize the unwanted components of the sequencing reactions following manufacturer’s protocols. Applied Biosystems 3730 DNA analyzer using a protocol with “extra long read length setting” was used to sequence *rpoS* gene using both forward and reverse primers. DNA sequences were analyzed manually for bases called incorrectly, trimmed at both 5' and 3' ends and assembled using Kodon (v. 3.5, Applied Maths, Inc., Austin, TX, USA).
